# J-shaped association of serum uric acid with all-cause and cardiovascular mortality in patients with diabetic kidney disease

**DOI:** 10.1007/s00592-024-02376-0

**Published:** 2024-11-14

**Authors:** Xinxin Zhang, Ziyue Zhang, Liyuan Gao, Bo Huang, Yue Liu, Jingqiu Cui, Junya Jia, Ming Liu

**Affiliations:** 1 Department of Nephrology, Tianjin Medical University General Hospital, 154 Anshan Road, Heping District, Tianjin, 300052 China; 2 Department of Endocrinology and Metabolism, Tianjin Medical University General Hospital, 154 Anshan Road, Heping District, Tianjin, 300052 China

**Keywords:** Serum uric acid, Mortality, Diabetic kidney disease, All-cause

## Abstract

**Aims:**

The association of serum uric acid (SUA) with mortality remains unclear in patients with diabetic kidney disease (DKD). Thus, this prospective cohort study aimed to explore the association of SUA with all-cause and cardiovascular disease (CVD) mortality among patients with DKD in a large, nationally representative sample.

**Methods:**

This cohort study included data from the National Health and Nutrition Examination Survey (NHANES) 1999–2018 and the National Death Index mortality data until 31 December 2019. The restricted cubic spline and the Cox proportional hazards regression were conducted to describe the association of SUA with all-cause and CVD mortality and evaluate potential nonlinear associations.

**Results:**

The analysis included 3470 patients with DKD from NHANES 1999–2018. During the follow-up time of 24,633 person-years, we recorded 1489 all-cause deaths, including 542 CVD deaths. We identified a J-shaped association of SUA with all-cause and CVD mortality. The corresponding inflection points were observed at *5.1* and 5.7 mg/dL. When SUA were higher than inflection points, each 1 mg/dL increase in SUA was linked to a 13% and 22% higher risk of all-cause (*HR: 1.13; 95% CI: 1.07–1.20;P < 0.001*) and CVD (*HR: 1.22; 95% CI: 1.06–1.41;P = 0.006*) mortality, respectively.

**Conclusions:**

This study indicated the J-shaped association of SUA with all-cause and CVD mortality in patients with DKD. The corresponding inflection points were *5.1* mg/dL for all causes and 5.7 mg/dL for CVD, respectively. More clinical randomized trials are needed to confirm the optimal uric acid-lowering target.

## Introduction

Diabetic kidney disease (DKD), a common microvascular complication among individuals with diabetes mellitus (DM), affects 25–40% of diabetic patients and stands as a major contributor of end-stage renal disease (ESRD) globally [1]. Diabetic patients with ESRD had a substantially higher mortality in comparison to non-diabetic ones [2]. Thus, identifying modifiable risk factors for DKD is crucial to avoid premature death and promote health outcomes.

Uric acid, the end product of purine metabolism in humans, is predominantly excreted through the kidneys and constitutes the cause of gout [3]. Previous research has indicated that hyperuricemia increases the risk of developing hypertension [4], cardiovascular disease [5, 6], DM [7], and chronic kidney disease (CKD) [5, 8]. Several epidemiological studies have explored the correlation between serum uric acid (SUA) and the likelihood of mortality in different populations. Among the general population of adults in the United States, there was a U-shaped association of SUA with all-cause, cardiovascular disease (CVD), and cancer mortality [9]. Moreover, higher SUA concentrations were found to be related to greater possibilities of death from all causes and CVD in individuals with diabetes [10]. Among patients with CKD, J-shaped relationships were also reported [11, 12] and the corresponding inflection points of SUA concentrations for all-cause and CVD mortality were 311.65 and 392.34 µmol/L, respectively [12]. Additionally, SUA was strongly correlated with the degree of proteinuria [13], and hyperuricemia predicted the progression of DKD in individuals with type 2 diabetes mellitus (T2DM) [14, 15]. The prevalence of hyperuricemia was higher in diabetic patients in comparison to general populations [16]. However, in patients with DKD, the association of SUA with mortality remains unclear and it is also unknown whether there is a threshold effect of SUA in this association.

Thus, this prospective cohort study aimed to explore the association of SUA with all-cause and CVD mortality among patients with DKD in a large, nationally representative sample.

## Methods

### Study population

The National Health and Nutrition Examination Survey (NHANES), conducted every 2 years by the National Center for Health Statistics (NCHS), is a multistage and nationally representative survey, which recruit individuals of non-institutionalized US civilians aged 2 months and older [17]. The survey evaluates nutrition and physical health through interviews and medical examinations. The study procedure received approval from the Ethics Review Board of the NCHS, and each participant provided signed informed consent.

**Fig. 1:**
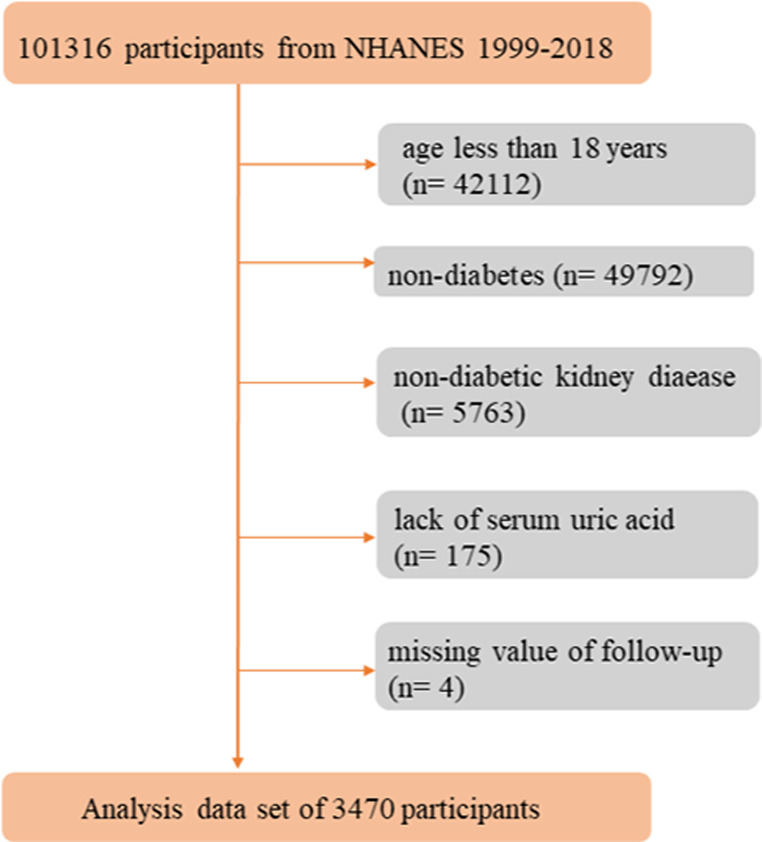
Flow chart of identification of study population. There were 3470 participants included in the present analysis

This cohort study included data from NHANES 1999–2018 [17] and the National Death Index (NDI) mortality data until 31 December 2019 [18]. Individuals meeting any of the following criteria were excluded: (1) participants less than 18 years; (2) participants without DKD; (3) missing information on SUA levels, and (4) information of death status was unavailable. Finally, the analysis included 3470 patients with DKD from NHANES 1999–2018 (Fig. 1).

### Measurement of SUA

The uricase method was used to measure SUA concentrations, with a measurable range of 11.9-1487.5 µmol/L. The reference intervals of adults were 202.3 to 416.5 µmol/L for men and 142.8 to 339.2 µmol/L for women.

### Outcomes

The determination of mortality due to all causes and CVD was conducted using the Public-Use Linked Mortality File until December 31, 2019 [18], which was connected to the NDI data. NDI is a centralized database managed by the NCHS, encompassing all recorded deaths in the United States. The cause of death is identified using the 10th Revision of International Statistical Classification of Diseases. CVD mortality refers to death caused by heart illnesses (coded as I00–I09, I11, I13, I20–I51) and stroke (coded as I60–I69). The duration of follow-up for individuals was determined by subtracting the baseline examination date from the latest recorded mortality status provided by the NDI data.

### Covariates

The data presented herein was gathered during home interviews using standardized questionnaires: demographic information, such as age, sex, ethnicity, education, and family income, along with details about physical activity, smoking and drinking status, *duration of diabetes*, and the use of antihyperuricemic agents (allopurinol and febuxostat). Anthropometric data, including body weight, height, and blood pressure was collected through physical examinations conducted at a mobile examination center (MEC). Blood samples were gathered for the measurements of lipid profiles, fasting blood glucose (FBG), glycosylated hemoglobin (HbA1c), and creatinine. Urine albumin and creatinine, obtained from urine samples, were used to calculate the urine albumin-to-creatinine ratio (ACR).

Participants were categorized as never smokers, former smokers, or current smokers based on their current smoking status and whether they had smoked ≥ 100 cigarettes in their lifetime. Drinking status was categorized into non-drinkers, former drinkers, or current drinkers due to their current drinking status and whether they had drunk more than or equal to twelve drinks in a lifetime. Body Mass Index (BMI, Kg/m^2^) was calculated by dividing the weight by the square of the height. The definition of obesity is a BMI ≥ 30 Kg/m^2^. The estimated glomerular filtration rate (eGFR) was determined by using the Chronic Kidney Disease Epidemiology Collaboration equation [19].

The definition of DM included a FBG level ≥ 7.0 mmol/L, a 2-hour plasma glucose level ≥ 11.1 mmol/L, an HbA1c level ≥ 6.5%, a self-reported DM history, or the use of hypoglycemic medications [20]. DKD was diagnosed when the ACR exceeded 30 g/g or the eGFR declined below 60 mL/min/1.73 m^2^ [21]. Dyslipidemia was diagnosed when meeting one or more of the following criteria: a total cholesterol level at least 5.18 mmol/L, a triglyceride level at least 1.7 mmol/L, a low-density lipoprotein cholesterol level at least 3.37 mmol/L, a high-density lipoprotein cholesterol level less than 1.04 mmol/L for males and 1.30 mmol/L for females, or the use of specific drug treatment. Hypertension was diagnosed by systolic blood pressure (SBP) ≥ 140 mmHg, diastolic blood pressure (DBP) ≥ 90 mmHg, a self-reported hypertension history, or the use of specific antihypertensive medications [22]. CVD was characterized by a medical background of congestive heart failure, coronary heart disease, angina, or stroke.

### Statistical analyses

The sample weight, representing the stratified, multistage design of the NHANES, was derived by multiplying 1/5 by the 4-Year MEC Weight (cycles from 1999 to 2002) or 1/10 by the 2-Year MEC Weight (cycles from 2003 to 2018). Categorical variables are displayed as numbers and percentages, whereas continuous ones are shown as means ± standard error (SE). Groups were compared using chi-square tests and one-way analysis of variance for categorical and continuous variables, respectively. Weighted Cox proportional hazards regression was conducted to explore the associations of SUA with all-cause and CVD mortality in patients with DKD after adjustment for confounders. SUA concentrations were also equally divided into tertiles, with the lowest tertile serving as a reference.

The restricted cubic spline along with the Cox proportional hazards regression was undertaken to describe the effect of SUA on all-cause and CVD mortality and evaluate potential nonlinear associations. If a nonlinear association was identified, a recursive algorithm would be applied to deduce the inflection points. Furthermore, piecewise Cox proportional hazard regression would be used to investigate the threshold effect.

Subgroup analyses were conducted within the following categories: age (*<* 65 y, ≥ 65 y), sex (women, men), obesity (no, yes), hypertension (no, yes), dyslipidemia (no, yes), CVD (no, yes), ACR (*<* 30 mg/g, ≥ 30 mg/g) and eGFR (*<* 60 mL/min/1.73 m^2^, ≥ 60 mL/min/1.73 m^2^). Due to the small population, the subgroup analysis of SUA with CVD mortality was not conducted. A two-sided P-value of less than 0.05 was used to determine statistical significance. The statistical analyses were performed using R software (version 4.2.2).

## Results

### Baseline characteristics

Table 1 illustrates the baseline characteristics of individuals by tertiles of SUA. Among 3470 DKD individuals from the NHANES dataset 1999–2018, the mean age was 64 years, and females accounted for 48.82%. Participants in higher tertiles of SUA were more likely to be older, male, obese, and have hypertension and CVD compared with those in the first tertile (all *P* < 0.001). Patients with higher SUA concentrations were related to lower diastolic blood pressure (DBP), high-density lipoprotein cholesterol, FBG, HbA1c, and eGFR and higher ACR (all *P* < 0.010). In addition, participants in the higher tertiles were less likely to use antihyperuricemic agents and smoke than those in the first tertile of SUA (all *P* < 0.05).

**Table 1:** Characteristics of participants categorized by tertiles of serum uric acid

Variables	Total	Serum uric acid, mg/dL			*P* value
		Tertile 1 (≤ 5.3 mg/dl)	Tertile 2 (5.4-6.8 mg/dL)	Tertile 3 (≥ 6.9 mg/dl)	
Participants	3470	1195	1156	1119	-
Age, years	64.25 ± 0.35	62.33 ± 0.48	64.17 ± 0.59	66.29 ± 0.62	< 0.001
Sex, %					< 0.001
Women	1633 (48.82)	641 (55.44)	520 (47.30)	472 (43.59)	
Men	1837 (51.18)	554 (44.56)	636 (52.70)	647 (56.41)	
Ethnicity, %					< 0.001
Non-Hispanic White	1374 (62.45)	415 (59.30)	476 (63.13)	483 (64.99)	
Non-Hispanic Black	855 (14.68)	238 (12.69)	274 (14.37)	343 (17.04)	
Mexican American	675 (8.94)	334 (13.31)	217 (8.87)	124 (4.52)	
Other	566 (13.93)	208 (14.70)	189 (13.62)	169 (13.46)	
Education, %					0.442
Some college or above	1249 (43.34)	395 (41.40)	435 (44.86)	419 (44.19)	
High school graduate	773 (25.86)	264 (27.21)	245 (23.72)	264 (26.90)	
Less than 12th	1428 (30.49)	528 (31.39)	471 (31.42)	429 (28.91)	
Smoking, %					< 0.001
never	1624 (46.24)	576 (46.54)	562 (49.40)	486 (43.12)	
former	1305 (38.00)	400 (34.56)	412 (35.43)	493 (44.51)	
now	525 (15.47)	212 (18.90)	178 (15.17)	135 (12.37)	
Drinking, %					0.669
never	622 (16.66)	230 (19.97)	206 (17.75)	186 (17.82)	
former	999 (25.89)	347 (28.16)	325 (27.68)	327 (30.48)	
now	1445 (47.46)	467 (51.87)	504 (54.57)	474 (51.70)	
Physical activity, %					0.246
no	1645 (44.57)	537 (42.86)	544 (43.63)	564 (47.28)	
yes	1825 (55.43)	658 (57.14)	612 (56.37)	555 (52.72)	
Family income-poverty ratio, %					0.825
≤ 1	788 (17.17)	298 (19.40)	250 (17.51)	240 (19.26)	
1–3	1566 (43.70)	532 (47.45)	526 (47.10)	508 (48.31)	
> 3	773 (30.91)	245 (33.15)	281 (35.39)	247 (32.43)	
Hypertension, %	2823 (80.44)	911 (74.85)	933 (78.88)	979 (87.80)	< 0.001
Dyslipidemia, %	3090 (90.01)	1053 (88.50)	1011 (89.26)	1026 (92.32)	0.058
Cardiovascular disease, %	1238 (34.66)	363 (30.09)	391 (31.99)	484 (42.24)	< 0.001
Use of antihyperuricemic agents, %	186 (5.40)	74 (7.41)	58 (4.48)	54 (4.29)	0.017
Duration of diabetes					0.201
≤ 5	678 (20.06)	230 (18.85)	233 (21.06)	215 (20.30)	
5–10	521 (15.55)	184 (17.37)	200 (16.26)	137 (12.95)	
10–15	436 (12.27)	160 (11.72)	130 (11.13)	146 (14.00)	
> 15	949 (26.01)	329 (26.66)	296 (23.53)	324 (27.85)	
Missing data	886 (26.11)	292 (25.39)	297 (28.03)	*297 (24.89)*	
BMI, Kg/m^2^	32.64 ± 0.21	30.93 ± 0.33	32.96 ± 0.32	34.12 ± 0.36	< 0.001
Blood pressure, mmHg					
Systolic	136.06 ± 0.54	136.99 ± 1.01	136.79 ± 0.85	134.35 ± 0.93	0.079
Diastolic	68.73 ± 0.40	70.03 ± 0.59	69.53 ± 0.65	66.58 ± 0.67	< 0.001
TC, mmol/L	4.91 ± 0.03	4.98 ± 0.07	4.89 ± 0.05	4.85 ± 0.06	0.336
TG, mmol/L	2.15 ± 0.07	2.07 ± 0.12	2.08 ± 0.09	2.29 ± 0.17	0.511
HDL-C, mmol/L	1.24 ± 0.01	1.29 ± 0.03	1.24 ± 0.02	1.19 ± 0.01	0.003
LDL-C, mmol/L	2.69 ± 0.03	2.68 ± 0.06	2.68 ± 0.05	2.70 ± 0.06	0.984
FBG, mmol/L	8.93 ± 0.11	9.78 ± 0.21	8.68 ± 0.20	8.39 ± 0.18	< 0.001
HbA1c, %	7.41 ± 0.04	7.89 ± 0.09	7.22 ± 0.06	7.10 ± 0.06	< 0.001
ACR, mg/g	317.11 ± 19.86	242.24 ± 26.14	301.68 ± 27.26	410.38 ± 42.89	0.002
eGFR, ml/ (min*1.73m^2^)	68.97 ± 0.70	79.90 ± 1.03	69.56 ± 1.21	57.15 ± 1.09	< 0.001

BMI, body mass index; TC, total cholesterol; TG, triglycerides; HDL-C, high density lipoprotein cholesterol; LDL-C, low density lipoprotein cholesterol; FBG, fasting blood glucose; HbA1c, glycosylated hemoglobin; eGFR, estimated glomerular infiltration rate; ACR, albumin to creatinine ratio

### Association of SUA with all-cause and CVD mortality

**Fig. 2:**
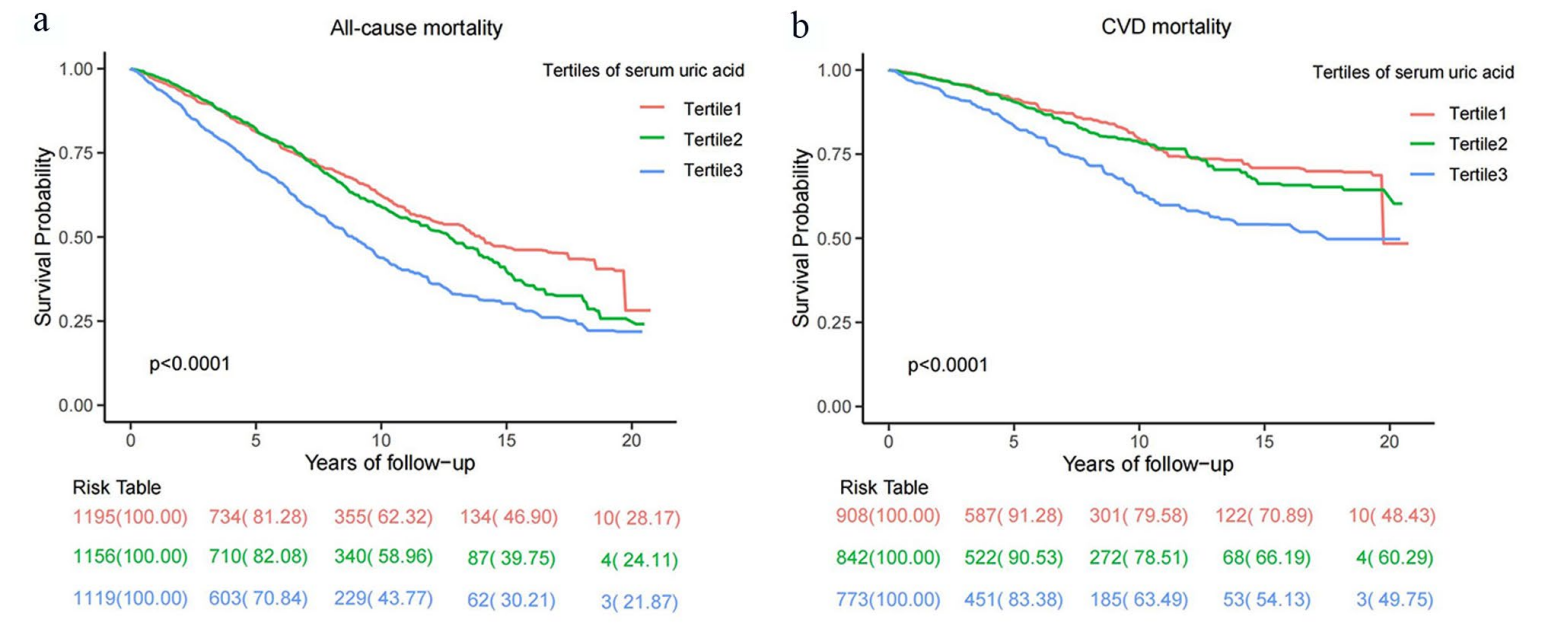
Kaplan-Meier survival curves showing mortality by tertiles of SUA. Participants in higher tertiles of SUA concentrations were related to increased risks of all-cause and CVD mortality

During the follow-up period of 24,633 person-years (median, 9.9 years), we recorded 1489 all-cause deaths from the NHANES 1999–2018, including 542 CVD deaths. Figure 2 shows all-cause and CVD mortality by tertiles of SUA using Kaplan-Meier survival curves. Participants in higher tertiles of SUA concentrations were related to increased risks of all-cause and CVD mortality (all *P* < 0.001, log-rank test).

The hazard ratios (HRs) for the association of SUA with all-cause and CVD mortality in three models are presented in Table 2. After adjustment for age, sex, BMI, ethnicity, education, family income-poverty ratio, smoking and drinking status, physical activity, dyslipidemia, hypertension, CVD, *duration of diabetes*, HbA1c, ACR, eGFR and the use of antihyperuricemic medications in model 3, the risk of all-cause mortality increased with an increasing SUA level; the adjusted HR and 95% confidence interval (CI) for tertile3 versus tertile1 was *1.32 (1.06–1.65;P = 0.013 for trend).* Each 1 mg/dL increase of SUA was also related to increased risks of mortality from all causes *(HR: 1.10; 95% CI: 1.04–1.16;P < 0.001)* and CVD (*HR: 1.12; 95% CI: 1.02–1.24;P = 0.017*). However, we didn’t find a significant correlation between SUA and CVD mortality when DHEA was divided into tertiles (*HR: 1.27; 95% CI: 0.89–1.81;P = 0.171 for the trend*).

**Table 2:** Association of SUA concentration with mortality in patients with DKD from the NHANES 1999–2018

	No. of participants	No. of deaths	Model 1			Model 2			Model 3	
			HR (95% CI)	*P* value		HR (95% CI)	*P* value		HR (95% CI)	*P* value
**All-cause mortality**										
Tertile 1	1195	447	Reference	-		Reference	-		Reference	-
Tertile 2	1156	479	1.13 (0.96,1.33)	0.141		1.02 (0.84,1.23)	0.875		*1.09 (0.89*,*1.34)*	*0.411*
Tertile 3	1119	563	**1.69 (1.43**,**2.00)**	**< 0.001**		**1.29 (1.05**,**1.58)**	**0.016**		**1.32 (1.06**,**1.65)**	**0.014**
P for trend				**< 0.001**			**0.014**			**0.013**
Per 1 mg/dL increment	3470	1489	**1.16 (1.12**,**1.20)**	**< 0.001**		**1.10 (1.04**,**1.16)**	**< 0.001**		**1.10 (1.04**,**1.16)**	**< 0.001**
**CVD mortality**										
Tertile 1	908	160	Reference	-		Reference	-		Reference	-
Tertile 2	842	165	1.10 (0.85,1.42)	0.477		0.91 (0.67,1.25)	0.912		*0.98 (0.68*,*1.43)*	*0.923*
Tertile 3	773	217	**1.89 (1.46**,**2.44)**	**< 0.001**		1.28 (0.94,1.75)	0.282		*1.27 (0.89*,*1.81)*	*0.196*
P for trend				**< 0.001**			0.092			*0.171*
Per 1 mg/dL increment	2523	542	**1.22 (1.15**,**1.30)**	**< 0.001**		**1.12 (1.03**,**1.23)**	**0.009**		**1.12 (1.02**,**1.24)**	**0.017**

Model 1: Unadjusted

Model 2: adjusts for age, sex, BMI, ethnicity, education, family income-poverty ratio, smoking and drinking status, and physical activity

Model 3: adjusts for model 2 + dyslipidemia, hypertension, CVD, duration of diabetes, HbA1c, ACR, eGFR and the use of antihyperuricemic agents

DKD, diabetic kidney disease; CI, confidence interval; HR, hazard ratio; BMI body mass index; CVD, cardiovascular disease; HbA1c, glycosylated hemoglobin; eGFR, estimated glomerular infiltration rate; ACR, albumin to creatinine ratio

**Fig. 3:**
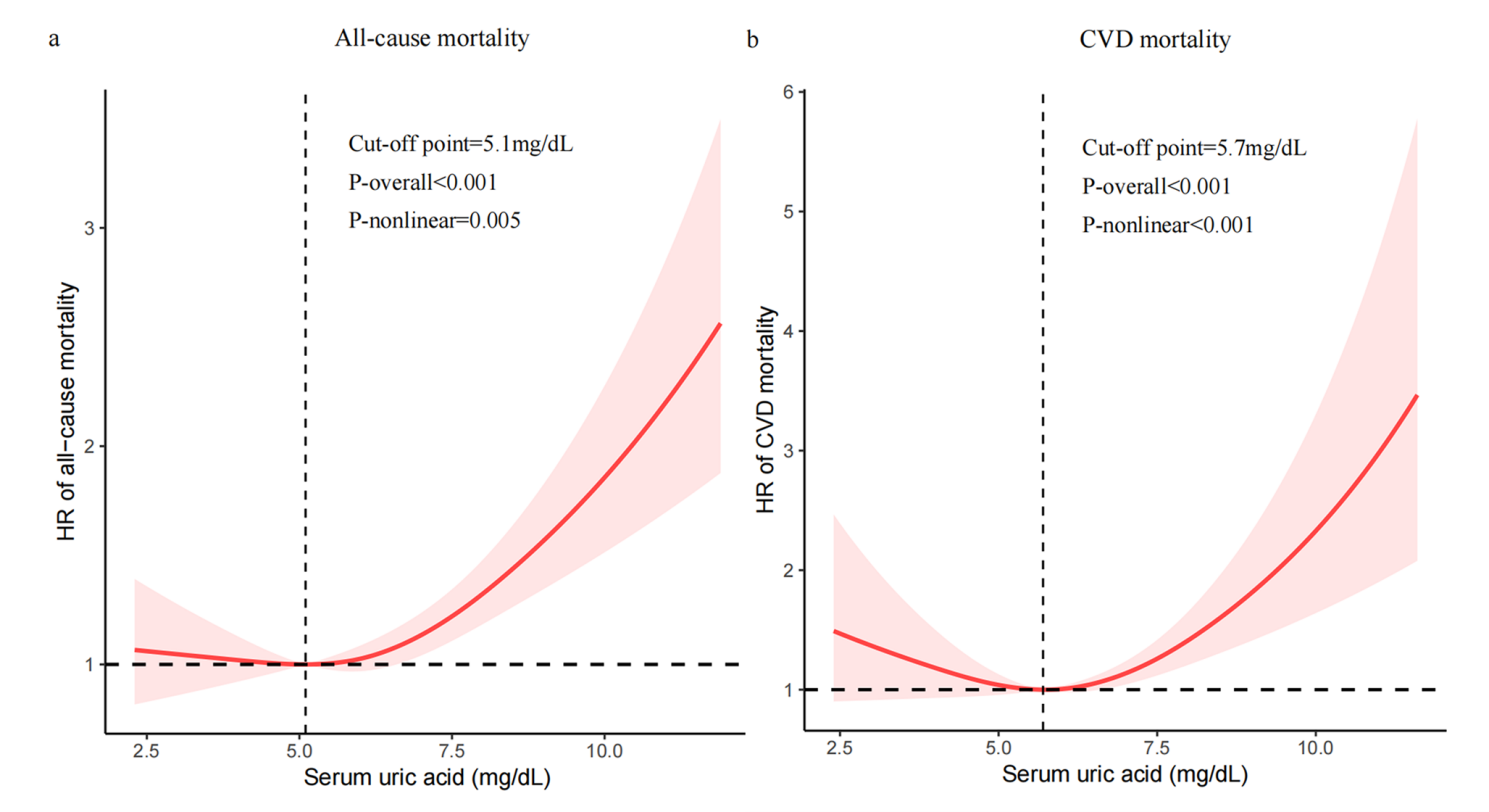
The J-shaped association of SUA with all-cause and CVD mortality. The line indicates the adjusted HRs and 95% CI is shown by shaded areas. Adjusted for age, sex, BMI, ethnicity, education, family income-poverty ratio, smoking and drinking status, physical activity, dyslipidemia, hypertension, CVD, duration of diabetes, HbA1c, ACR, eGFR and the use of antihyperuricemic agents. The inflection points were *5.1* mg/dL and 5.7 mg/dL for all-cause and CVD mortality, respectively

Figure 3 describes J-shaped nonlinear associations of SUA with all-cause(Figure3a) and CVD(Figure3b) mortality in individuals with DKD. The solid line indicates the adjusted HRs and 95% CI is shown by shaded areas. The vertical dashed line presents the inflection points of SUA.We found that the inflection points were *5.1* mg/dL and 5.7 mg/dL for all-cause and CVD mortality, respectively. As shown in Table 3, when SUA levels were higher than inflection points, per 1 mg/dL increment in SUA was linked to a 13% higher risk of all-cause mortality (*HR: 1.13; 95% CI: 1.07–1.20;P < 0.001*) and 22% higher risk of CVD mortality (*HR: 1.22; 95% CI: 1.06–1.41;P = 0.006*)."-->

**Table 3:** Threshold effect analysis of serum uric acid on mortality in patients with DKD from the NHANES 1999–2018

Serum uric acid, mg/dL	HR (95% CI)	*P* value
**All-cause mortality**		
Weighted cox regression model	**1.10 (1.04**,**1.16)**	**< 0.001**
Weighted two-piecewise cox regression model, Cut-off point, 5.1 mg/dL		
< 5.1 mg/dL	1.03 (0.81,1.31)	0.790
≥ 5.1 mg/dL	**1.13 (1.07**,**1.20)**	**< 0.001**
**CVD mortality**		
Weighted cox regression model	**1.12 (1.02**,**1.24)**	**0.017**
Weighted two-piecewise cox regression model, Cut-off point, 5.7 mg/dL		
< 5.7 mg/dL	*1.04 (0.78*,*1.40)*	*0.789*
≥ 5.7 mg/dL	**1.22 (1.06**,**1.41)**	**0.006**

Adjusted for age, sex, BMI, ethnicity, education, family income-poverty ratio, smoking and drinking status, physical activity, dyslipidemia, hypertension, CVD, duration of diabetes, HbA1c, ACR, eGFR and the use of antihyperuricemic agents

DKD, diabetic kidney disease; CI, confidence interval; HR, hazard ratio; CVD, cardiovascular disease

### Subgroup analyses of the relationship between SUA and all-cause mortality

**Fig. 4:**
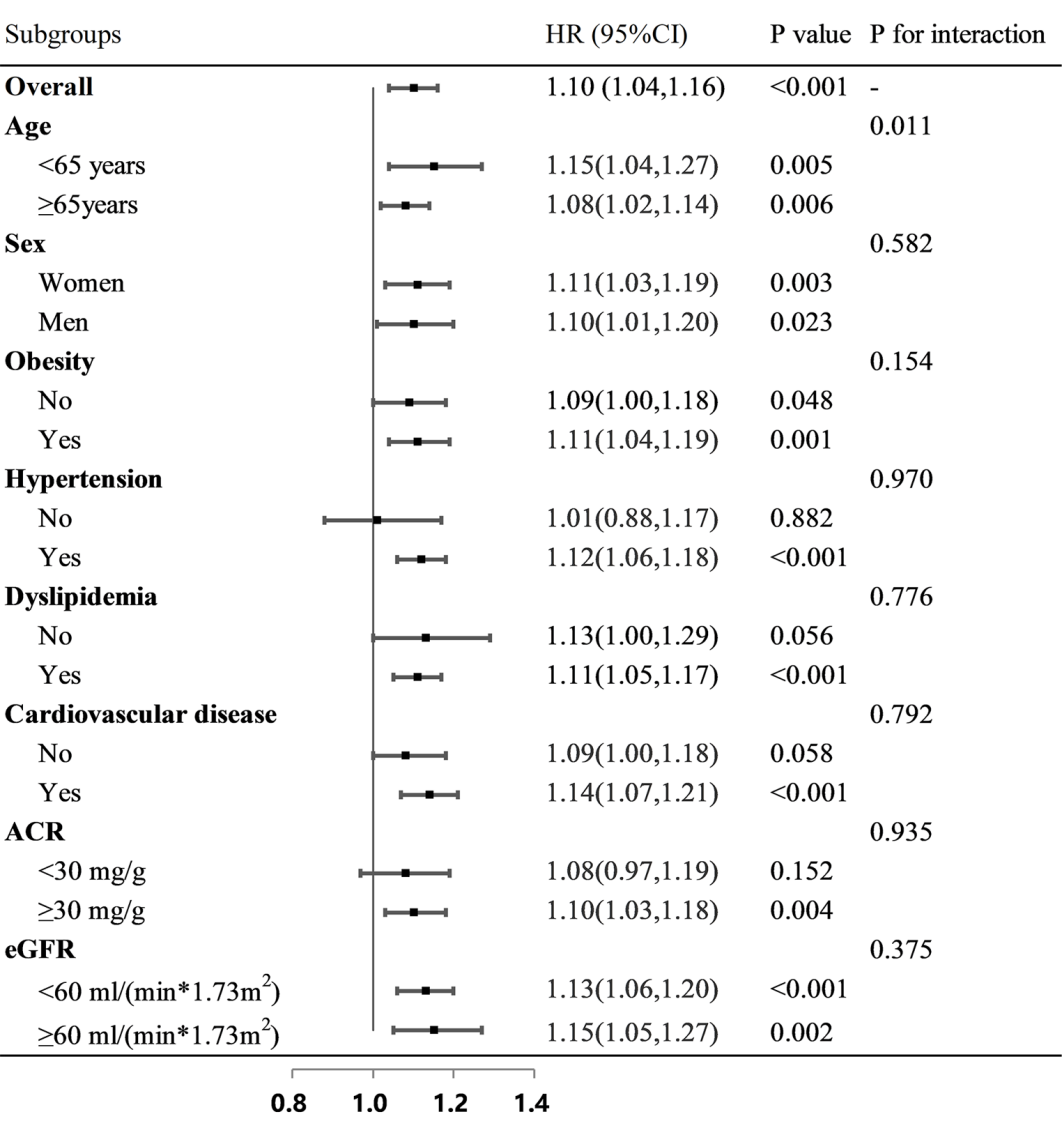
Subgroup analyses of the associations of SUA with all-cause mortality. Adjusted for age, sex, BMI, ethnicity, education, family income-poverty ratio, smoking and drinking status, physical activity, dyslipidemia, hypertension, CVD, duration of diabetes, HbA1c, ACR, eGFR and the use of antihyperuricemic agents

Figure 4 presents the association of SUA with all-cause mortality in different subgroups. The effect of SUA on all-cause mortality was stable in the sex, obesity, hypertension, dyslipidemia, CVD, ACR and eGFR subgroups (all P for interaction > 0.05). Tests for interaction showed that this association was statistically different in age subgroups (*P for interaction = 0.011*). In participants aged < 65 years, SUA had a more remarkably increasing risk of all-cause mortality than in those ≥ 65 years (*< 65 years*,* HR: 1.15; 95% CI: 1.04–1.27;P = 0.005; ≥65 years*,* HR: 1.08; 95% CI: 1.02–1.14;P = 0.006*).

## Discussion

Based on data from NHANES 1999–2018, we investigated the association of SUA with all-cause and CVD mortality in a large, nationally representative sample of patients with DKD. We found that there was a J-shaped association of SUA with all-cause and CVD mortality and that, when SUA levels were higher than *5.1* and 5.7 mg/dL, per 1 mg/dL increase in SUA was related to a 13% and 22% higher risk of all-cause and CVD mortality, respectively. Subgroup analyses illustrated that, in participants aged < 65 years, SUA had a more remarkably increasing risk of all-cause mortality than in those ≥ 65 years.

The correlation between SUA and mortality has been investigated in many previous studies, the results of which were controversial. The NHANES 1999–2002 including 9118 US adults proved that both low and high SUA concentrations were related to a higher chance of all-cause and CVD mortality and that the threshold level of SUA was 6 mg/dL in men and 4 mg/dL in women, respectively [9]. Moreover, a U-shaped relationship was also found between SUA and CVD mortality in a Japanese community-based study involving residents aged 40 years and older [23]. Data from the Irish health system revealed that the association between SUA and all-cause mortality was U-shaped in men and J-shaped in women; optimal SUA levels were identified as 5.1–7.6 mg/dL and < 6.9 mg/dL for men and women, respectively [24].

Furthermore, there were also no consistent conclusions about this association for individuals with diabetes. The NHANES 1999–2018 and subsequent meta-analysis proved that higher levels of SUA were related to increased risk of deaths from all causes and CVD [10]. However, three cohorts of patients with T2DM reported that, patients with relatively higher and lower levels had an increased risk of all-cause mortality in comparison to those with moderate SUA levels [25].

A systematic review and meta-analysis revealed that individuals with CKD have an 8% higher likelihood of death from all causes for every 1 mg/dL increase in SUA levels [26]. Additionally, a prospective cohort study including CKD patients from NHANES 1999–2018 found that there were J-shaped associations of SUA with mortality and that the corresponding inflection points were 5.2 mg/dL for all causes and 6.6 mg/dL for CVD, respectively [12]. In large dialysis cohort studies, a U-shaped correlation was also proved between SUA and both all-cause and CVD mortality [27, 28]. The differences in study populations, sample sizes, follow-up durations, and adjusted covariates may partially account for the variations in the conclusions mentioned above.

The variability of SUA has also proved to be associated with health outcomes. A retrospective cohort study including 44,176 healthy participants found that, regardless of the baseline SUA level, the longitudinal increase in SUA was associated with an increased risk of developing metabolic syndrome [29]. Moreover, the increase of SUA was also related to higher risks of T2DM and renal function decline [30, 31]. The Kailuan Study proved that higher variability of SUA was identified to increase the risk of death from all causes regardless of the direction of variability and that the optimal range was deemed to be within 20% [32, 33].

In present study including participants with DKD, we identified a J-shaped association of SUA with all-cause and CVD mortality. The corresponding inflection points of SUA concentrations were observed at *5.1* mg/dL for all causes and 5.7 mg/dL for CVD, respectively. Perhaps the SUA value of 5.7 mg/dl is the intervention target of uric acid-lowering therapy in DKD patients with high-risk factors for CVD mortality. The nonlinear association mentioned above can be explained by the complex physiology of uric acid. On the one hand, SUA may act as a potent antioxidant in extracellular environments [34, 35], which plays a protective role in CVD, aging, and cancer [36]. On the other hand, SUA at supraphysiological levels may act as a pro-potent antioxidant [34]. Both clinical studies and cell experiments have confirmed that elevated SUA levels contribute to endothelial dysfunction by downregulating nitric oxide and increasing mitochondrial reactive oxygen species [37–40]. It should be noted that there is no simple explanation for the potential protective or pathogenic effects of SUA, and further animal experiments are needed to investigate this association. In addition, hyperuricemia has been considered a risk factor for adverse complications, such as hypertension, CVD, and metabolic disease [3], which may contribute to the relationship between SUA and mortality.

We also found that, in participants aged < 65 years, SUA had a more remarkably increasing risk of death from all causes in comparison to those ≥ 65 years. There are several possible reasons to explain this finding: Firstly, younger individuals may be more susceptible to the influence of uric acid levels because their physiological conditions and metabolism are likely more active. Secondly, in the older population, the impact of other chronic diseases on the risk of mortality may be greater, while the role of uric acid is relatively smaller. More research in the future is needed to explain this phenomenon.

Despite the negative impact of high SUA levels on health outcomes, therapeutic outcomes of reducing uric acid remain controversial. In a multicenter, prospective, randomized clinical trial, 514 asymptomatic hyperuricemia patients were randomly assigned to dose-titrated febuxostat (10–60 mg per day, *n* = 239) or control group (lifestyle changes for hyperuricemia, *n* = 244) [41]. This clinical trial found that, compared to non-pharmacological treatment, febuxostat treatment for 24 months did not delay the progression of carotid atherosclerosis [41]. In addition, a comprehensive meta-analysis showed that uric acid-lowering administration did not improve the prognosis amomg heart failure patients [42]. *A recent study demonstrated that lowering SUA levels in diabetic patients had no impact on renal outcomes* [43]. *This raises the possibility that reducing SUA might also not affect all-cause and cardiovascular disease mortalities.* However, in a prospective, randomized study performed across 141 hospitals, a total of 1070 patients were recruited and randomly assigned to the febuxostat and the non-febuxostat groups. The optimal SUA level of 5–6 mg/dl can reduce all-cause mortality, as well as cardiovascular, and renal events [44, 45]. *Given the complex role of SUA as both an antioxidant and a contributor to endothelial dysfunction*,* we speculate that simply lowering SUA without considering its physiological role might not be beneficial.* Therefore, more clinical randomized trials are warranted to confirm the effect of uric acid-lowering therapy on health and the optimal intervention target range of SUA levels.

This study had several limitations. First, the observational nature of the current analysis hinders our ability to establish a causal association of SUA with mortality. Second, because SUA concentrations were measured only one single time in the current population, we are unable to explore the variability of SUA with mortality. Third, due to the small population of our study, the sex differences of SUA with mortality were not conducted in this present analysis. Fourth, the NHANES data cannot exclude patients who simultaneously have non-diabetic kidney disease. Finally, despite adjustment for numerous potential confounders, the presence of residual confounders cannot be completely eliminated.

In conclusion, we found that there was a J-shaped association of SUA with all-cause and CVD mortality in patients with DKD and that the corresponding inflection points were observed at *5.1* mg/dL and 5.7 mg/dL for deaths from all causes and CVD, respectively. These results indicate that maintaining an appropriate SUA concentration may have a positive impact on the long-term health outcomes among patients with DKD. More clinical randomized trials are needed to confirm the optimal uric acid-lowering target.

## Data Availability

The data that support the findings of this study are openly available online (https://wwwn.cdc.gov/nchs/nhanes/default.aspx).
